# Epigenetic modulation in tuberculosis – mycobacterial defense mechanism to host immune responses

**DOI:** 10.3389/fimmu.2026.1943755

**Published:** 2026-09-01

**Authors:** Karthikeyan Sundaram, Sridhar Rathinam, Ramalingam Bethunaickan, Uma Devi Ranganathan, Madhavan Dhanapal, Harish Selvaraj, Venkataraman Prabhu

**Affiliations:** 1 Department of Herbal Pharmacology and Environmental Sustainability, Chettinad Hospital and Research Institute, Chettinad Academy of Research and Education, Kanchipuram, Tamil Nadu, India; 2 Chettinad Hospital and Research Institute, Chettinad Academy of Research and Education, Kanchipuram, Tamil Nadu, India; 3 Department of Immunology, Indian Council of Medical Research (ICMR)- National Institute for Research in Tuberculosis, Chennai, Tamilnadu, India; 4 Faculty of Medical Research, Academy of Scientific and Innovative Research (AcSIR), Ghaziabad, India; 5 Division of Medical Research, SRM Medical College Hospital and Research Centre, Chennai, Tamil Nadu, India

**Keywords:** acetylation, epigenetics, histone modification, methylation, non-coding RNA, tuberculosis

## Abstract

Epigenetic regulators alter chromatin to control transcription in response to pathogens. Histone acetylation influences DNA accessibility and gene expression, impacting latent and active tuberculosis (TB). *Mycobacterium tuberculosis* (MTB) requires specific components to harm the host immune system. The review aims to analyze MTB epigenetic processes and the impact of host immune system genes. This review analyzed histone deacetylases, DNA methyltransferases, epigenetic linkages to non-coding RNAs, and enhanced epigenetic detection. Also, it described mycobacteria’s evasion of the host immune system through lipid metabolism, autophagy, oxidative stress, and epigenetic efflux pump inhibitors. The MTB DNA methyltransferase (DNMT) *Rv2966c* (I6XFS7) modifies histones H3 and H4, impacting host transcription. The MTB acetyltransferase EIS (*Rv2416c*, P9WFK7) enhances human IL-10 synthesis and acetylates the H3 gene promoter. MTB infection activates an epigenetic repressor complex with HDAC1 and ZBTB25, which restricts IL-12B production in macrophages. MTB inhibits HDAC1 and ZBTB25, enhances autophagic clearance, reduces bacterial viability, and increases IL-12B. ADP-ribosyl polymerase *Rv1899c* interacts with HDAC1 and may inhibit immune activation. *Rv1899c* interacts with HDAC1 and ZBTB25 to silence inflammatory promoters and maintain immune suppression without effector release. NLRP3 activation is influenced by DNA methylation. DNMT inhibitor DAC increased transcriptional activity *in vitro*, while DNA methylase Sss I decreased NLRP3 promoter activity. In small RNAs, links to epigenetics, *the miR-15/16* family’s *miR-15a-5p*, regulate apoptosis, the cell cycle, and inflammatory cytokines. *MiR-15a-5p* affects inflammation by targeting genes in the BCL2, IKKα, and NF-κB pathways. Thus, epigenetic studies on MTB are vital to identify host immune evasion and host-directed therapy.

## 1 Introduction

Tuberculosis (TB) is an infectious disease and a top ten death-associated infection caused by the acid-fast bacilli *Mycobacterium tuberculosis* (MTB). During the infection, alveolar macrophages infected with MTB suppress phagosome formation by altering host signaling pathways to provide a niche for their survival (1). Alveolar macrophage activation alters production of chemokines and pro- and anti-inflammatory cytokines that regulate innate and adaptive immune responses. Epigenetic regulators regulate pathogen-induced transcription via altering chromatin. Histone acetylation regulates DNA accessibility and gene expression. Histone acetyltransferases (HATs) acetylate histone-tail lysines to relax chromatin and control transcription. Histone deacetylases (HDACs) inhibit HATs by removing acetyl groups from conserved lysine residues, compacting chromatin and inhibiting transcription (2). However, Khosla et al., 2016 noted that histone changes such arginine methylation in histone H3 make gene transcription dynamic during development. *Rv1988*’s H3R42 dimethylation lets mycobacteria control host gene expression (3).

Epigenetics investigates latent and active tuberculosis. MTB requires these molecules to influence host genes in the innate and adaptive immune systems. Recent research investigated the proteins of nine of these compounds. The histone acetyltransferase *Rv3423.1* (P9WKY5) from the Mycobacterial H37Rv strain influences the expression of host anti-inflammatory genes (4). The MTB DNA methyltransferase *Rv2966c* (I6XFS7) influences host transcription by modifying histones H3 and H4 (5). MTB’s acetyltransferase EIS (*Rv2416c*, accession number P9WFK7) upregulates human IL-10 expression. Acetylation of the H3 gene promoter. This protein facilitates the evasion of autophagy and sustains internal persistence (6). In addition, the Sir2-like protein *cobB* (*Rv1151c*, accession number: P9WGG3) influences DNA compaction in MTB. Deacetylate acetylated histone ubiquitin. EsxA, a 6 kDa protein, regulates the host’s immune response to infection and the cytoplasmic escape of MTB, serving as a potent human T-cell antigen (7, 8). Thus, the review aims to evaluate the various epigenetic mechanisms of *M. tuberculosis* and their impact on the alteration of host immune system genes.

## 2 Host epigenetic regulation

**Figure 1:**
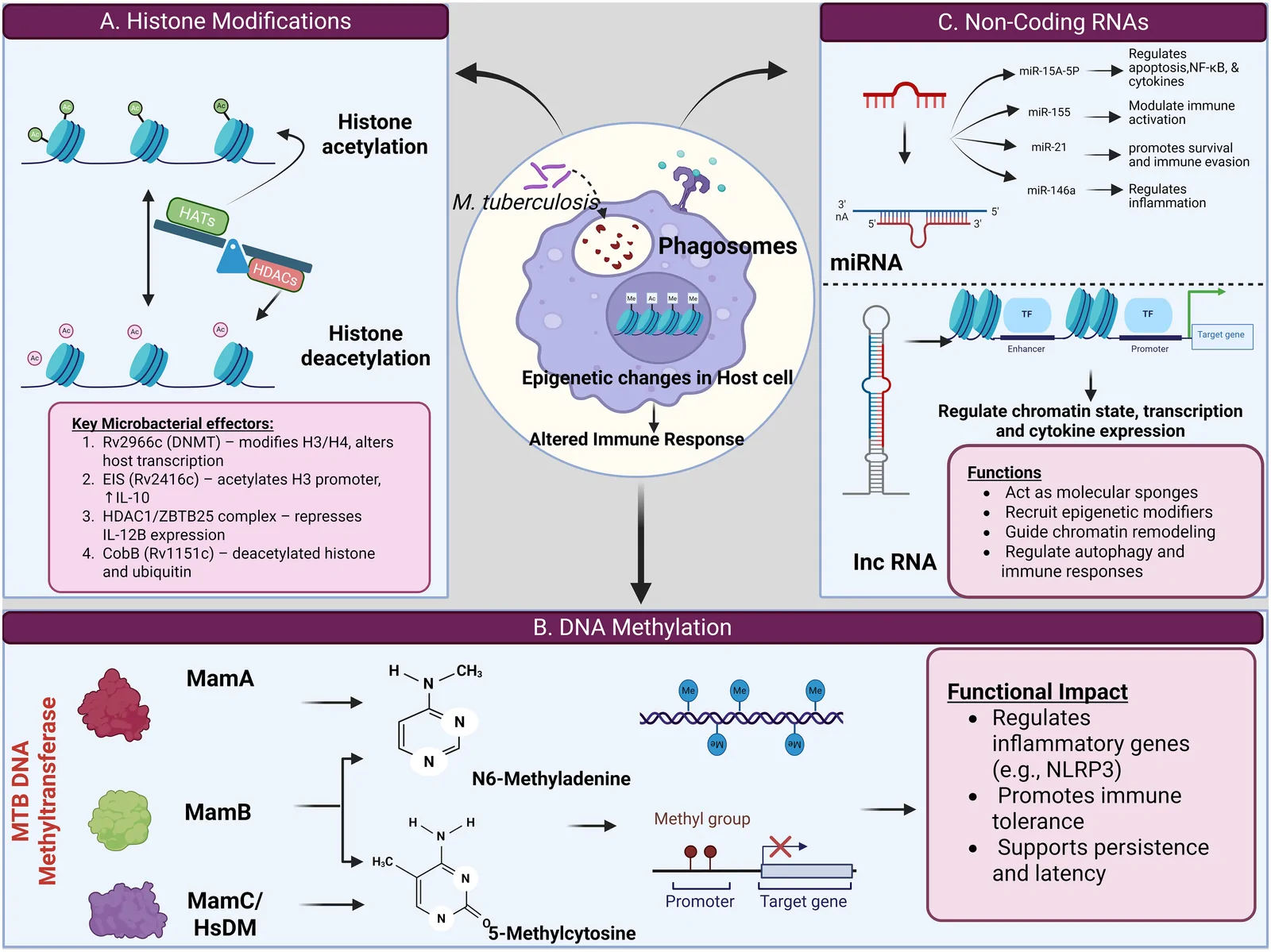
Mycobacterium tuberculosis modulates host immune responses through epigenetic mechanisms. A schematic representation of the main epigenetic processes that Mycobacterium tuberculosis (Mtb) uses to rewire host immunological responses. **(A)** Histone modifications that affect host chromatin accessibility and gene transcription, such as histone acetylation and deacetylation mediated by host histone acetyltransferases (HATs) and histone deacetylases (HDACs), as well as typical Mtb effectors. **(B)** Mycobacterial DNA methyltransferases (MamA, MamB, and MamC/HsdM) mediate DNA methylation, which results in N6-methyladenine and 5-methylcytosine alterations that control immunological tolerance, bacterial survival, and host inflammatory gene expression. **(C)** Non-coding RNA-mediated regulation, which includes long non-coding RNAs (lncRNAs) and microRNAs (miR-15a-5p, miR-155, miR-21, and miR-146a) that affect immunological signalling, autophagy, cytokine production, transcriptional control, and chromatin remodelling. By altering host transcriptional programs and reducing antimicrobial immunological responses, these epigenetic processes work together to support Mtb’s intracellular survival within macrophages.

Microbial pathogens utilize epigenetic regulators to control gene activity without changing the DNA sequence for protection. Infectious agents alter host epigenetics in immune cells, impacting gene transcription (**Table 1**). These changes result from their varied metabolites and byproducts. Examples are RNA modifications, histone modulation, chromatin remodeling, DNA methylation, and hydroxymethylation (15). However, studies indicate that HIV, MTB, and other pathogens utilize epigenetic mechanisms to modify host gene expression, leading to infection and proliferation in host cells. DNA methylation regulates many biological activities. Many DNA methyltransferases (DNMTs) methylate the genome. Cytosine’s fifth carbon gets methyl groups (–CH3) from the cofactor S-adenosyl-L-methionine in DNMTs. DNA methylation is a permanent epigenetic change. 5-methylcytosine (5mC) attracts histone deacetylases and tightens chromatin, extending transcriptional suppression (**Figure 1**) (16, 17).

**Table 1:** Different types of epigenetics modulation on MTB infections.

Study	Epigenetic/transcriptomic marker	Study outcome	Mechanism of action	Technical platform
Moreira et al., 2022 (9)	IL-1β; LPS/CD40LT; HDAC1	HDACs regulate gene transcription by removing acetyl groups from core histones. Over time, DHOB targeting HDAC1 led to increased IL-1β production, whereas Mtb-infected and LPS/CD40LT-stimulated DC showed considerably higher IL-1β mRNA levels than controls. None of the three inhibitors changed it. Mtb-infected dendritic cells stimulated with LPS/CD40LT showed much more pro-IL-1β expression than uninfected or stimulated controls. DHOB converted pro-IL-1β (31 kDa) to mature IL-1β (17 kDa), but the other three inhibitors did not influence production.	HDACs regulate gene transcription by deacetylating core histones, affecting IL-1β production at transcriptional, translational, and post-transcriptional stages.	IL-1β mRNA was quantified by real-time PCR at four- and sixteen-hours post-stimulation
Menon et al., 2025 (10)	*Rv1899c* (*MS_Rv1899c*) HDAC1–ZBTB25-interacting protein;	After 24 hours, *MS_Rv1899c*-infected macrophages release fewer pro-inflammatory cytokines (IL12B, IL-1β, and IL-6) and more anti-inflammatory cytokines (IL-10) than MS_Vec. Also, lower transcription of Beclin-1, which nucleates autophagosomes, and ATG5, which regulates their formation and flow, in *MS_Rv1899c*-infected macrophages. *MS_Rv1899c*-infected macrophages had reduced LC3-I to LC3-II conversion, indicating autophagosome inhibition.	*Rv1899c* reduces pro-inflammatory cytokines and autophagy and enhances anti-inflammatory cytokines to support mycobacterial survival.	qPCR and Western blot studies
Ahmadi Jeshvaghane et al., 2024 (11); Moopanar et al., 2023 (12)	MamCWT	The coverage depth’s peak-to-trough ratio (PTR) defines growth rate and divergence from the fitted regression line, which shows growth phase uniformity. Compared to their genetic background, MamC_WT_-anomalous isolates displayed a high PTR growth rate and a mostly consistent orientation axis coverage bias (lower MSE). MTB lineage 2 (East-Asian)’s Modern Beijing sublineage (L2.2.1) has all three MamC_WT_-anomalous isolates.	East-Asian isolates expanded slower than all other lineages except Euro-American, corroborating prior findings. On average, East-Asian isolates exhibited a lower PTR than Indo-Oceanic.	peak-to-trough ratio (PTR) analysis
Martini et al., 2023 (13)	sRNA MTS1338 (ncRv11733)	A logarithmic growth phase RNA-seq investigation of control and MTS1338-overexpressing cells. 10–19 million non-chimeric fragments were unambiguously mapped for each sample, although only 6% of reads matched to rRNA. The strain that overexpressed MTS1338 downregulated five genes and upregulated 55 genes. Also, MTS1338 overexpression elevated *Mpt70, Hsp*, and *ClpB* genes. After low pH exposure, both strains showed 33 DEGs, including stress response genes like the ethA/R region and metabolic pathways like fatty acid, methylcitrate, and propionate metabolism.	Differentially expressed genes (DEGs) encoded regulatory proteins or proteins involved in bacterial adaptability to harsh conditions, persistence, and infection. Also, Overexpression of *VapBC, mazEF, ArsR* metalloregulatory repressors, *CmtR*, and *CadI* increased metal ion homeostasis.	A logarithmic growth phase RNA-Seq investigation; Mycobrowser
Chen et al., 2025 (14)	LC3B; ATG5	Active TB patients with systemic symptoms (fever or weight loss) showed increased LC3B gene DNA methylation at the -63 CpG site (n = 35; 4.37 ± 3.51%) compared to those without symptoms (n = 25; 2.86 ± 2.17%, p = 0.03) or the NIHS with LTBI group (n = 46; 3.14 ± 2.2, p= 0.034) Active TB patients with systemic symptoms had higher DNA methylation levels at ATG5 gene sites (1.69 ± 0.37%, p < 0.001) and without (1.72 ± 0.34%, p = 0.016) compared to the NIHS plus LTBI group (1.52 ± 0.32%), which was negatively correlated with T-helper cells.	ESAT-6 stimuli did not alter ATG5 or LC3B gene promoter methylation, suggesting that hypermethylated LC3B gene promoter may represent an epigenotype for active TB rather than a modification acquired through chronic MTB infection.	DNA methylation in subgroup analysis; *In-vitro* assays

## 3 Mycobacterial epigenetic mechanism

Histone acetyltransferases (HATs) transfer acetyl groups from acetyl coenzyme A to histone N-terminal tails. Relaxed chromatin makes target gene regulatory units more accessible to transcriptional machinery, activating them. However, histone deacetylases (HDACs) remove acetyl groups from histone lysine residues, compacting chromatin for transcriptional control (9). HATs acetylate histones H3 and H4, enhancing gene transcription. Four types of histone deacetylases (HDACs) remove acetyl groups. Class I HDACs (1, 2, 3, and 8) are ubiquitously expressed, whereas Class II HDACs (4 and 5) exhibit tissue-specific expression. Histone deacetylase activity often inhibits gene expression (18).

HDACs are needed to make host defense genes like kinases, cytokines, transcriptional regulators, and pattern-recognition receptors (PAMPs). The role of HDACs in immune modulation is unclear, however pan-HDAC inhibitors boost the production of pro-inflammatory cytokines including IL-1β and other mediators (19, 20). Bacterial DNA methyltransferases (MTases) regulate gene expression, antiviral defense, and restriction modification (21). The DNA methyltransferases *MamA, MamB*, and *MamC* or *HsdM* modify m6A. MTB genomes exhibit N6-methyladenine (m6A) and 5-methylcytosine (m5C) methylation mechanisms. The well-characterized *MamA* methyltransferase specifically recognizes the sequence pattern CTCCAG. In hypoxic conditions, its absence can reduce gene expression and survival (22, 23). Consequently, three MTB MTases, including *MamC*, exhibit intracellular stochastic methylation. Chromohalobacter salexigens leads. In the second case, MTB DNA MTase *MamA* caused IMM in MamA_WT_ isolates after methionine deprivation, which was constitutive in some bacteria harboring *MamA* alleles. The novel evidence of direct transcriptional regulation by promoter *MamC* methylation shows that the *MamC* IMM shown here leads in heterogeneous gene expression and phenotypically distinct subpopulations. This greater phenotypic plasticity is only seen in active *MamC* strains. Therefore, active *MamC* strains may be more adaptive. Most East Asian strains have varied *MamA* and active *MamC*, which is intriguing (11). In addition, the manifestation of drug-efflux pumps will enhance primary TB drug resistance, especially the *Rv1258c* homologs. Modification of metabolic pathways, potential changes in mycolic acid synthesis during isoniazid stress. Immune evasion genes reduce the lethality of macrophages, DNA methylation serves to inhibit anti-drug resistance genes, and there is an alteration in bacterial stress responses. Phosphorylation in host signaling cascades (NF-kB, MAPK), phosphorylation of bacterial therapeutic targets (RNA polymerase subunits), and alteration of autophagy (24, 25).

### 3.1 DNA methyltransferases

The DNA methylation may cause MTB phenotypic variability, despite limited studies. MTB comprises *MamA, MamB*, and *HsdM* DNA MTases. Specific MTases methylate N6-adenine. Lineage-related loss-of-function (inactive) mutations are common in these genes. These little genetic changes may explain lineage-wide phenotypic variability via methylome diversity. They screened few isolates from each MTBC lineage, had no resistant isolates, and did not analyze motif kinetics. Also, the SMRT sequencing found methylation-free isolates. Notably, recent research compared allele-function mappings for the three known MTBC DNA adenine MTases to methylomic data and found improvements. To resolve MTase function inconsistencies, mamAE270A, mamAG152S, and mamBK1033T showed partial activity. Three alleles have been shown to generate mosaic methylation in 38 of 93 samples, including 34 of 36 EAS isolates. Comparing isolates’ methylomic data revealed hypomethylated and hypervariable motif locations. Function and integrative studies identified more methylated promoters, indicating DNA adenine methylation influences clinically relevant aspects. *HsdM* promoter methylation directly impacts transcription methylation, contrary to previous research. These findings support the growing evidence that genetic and phenotypic studies of microbial diversity, gene control, and evolution need bacterial epigenomics (26, 27).

Noticeably, the knocking out *hsdM* in BCG resulted in *ΔhsdM* exhibiting higher INH resistance compared to BCG, while complementary *HsdM* partially decreased INH resistance. *HsdM* substrates were enriched in the respiration pathway; *MamA* and *MamB* substrates were not. *HsdM*’s biological activity relates to redox homeostasis, supported by bioinformatic analysis of 25% of its substrates in the respiration pathway. bacterial GATC methylome investigations reveal MTases control transcription. In MTB, *HsdM* motifs exceed *MamA* and *SigA* motifs in ORFs, suggesting distinct biological roles. *HsdM* methylation occurs at 88% of ORF sites. Results show higher motif expression levels in *ΔhsdM*. Transcription of *HsdM*-methylated encoding genes may be affected (26–28).

Additionally, we must ascertain the impact of DNA methylation on the activation of NOD-like receptor (NLRP3). *In vitro*, the DNMT inhibitor DAC (5-aza-2′-deoxycytidine) enhanced transcriptional activity, but DNA methylase Sss I diminished NLRP3 promoter activity. This suggests that DNA methylation modulates NLRP3 expression and may exert physiological effects (29). Furthermore, Yan et al. posited that Omega-3 fatty acids mitigate metabolic problems and excessive inflammation by inhibiting the NLRP3 inflammasome. Dopamine regulates systemic inflammation by inhibiting NLRP3 inflammasome activity (30).

### 3.2 Histone deacetylases

An epigenetic repressor complex of HDAC1 and ZBTB25 limits macrophage IL-12B production during MTB infection. MTB manipulates host epigenetic mechanisms to evade protection by inhibiting HDAC1 and ZBTB25, which increased autophagic clearance, decreased intracellular bacterial viability, and restored IL-12B production (10). Similar research examined *Rv1899c*, an ADP-ribosyl polymerase that mostly interacts with HDAC1 and may directly inhibit immunological activation. *Rv1899c*’s association with HDAC1 and ZBTB25 is important because epigenetic silencing of inflammatory promoters sustains immune suppression without effector release. Bacteria hypoacetylate pro-inflammatory sites like IL-12 using HDAC1 to limit transcription, Th1 priming, and antimicrobial action (31).

Mycobacteria modify host cell gene expression through epigenetics. *Rv2966c* and *Rv1988* enable mycobacteria to interact directly with host chromatin, methylating host DNA cytosines and a specific non-tail arginine in histone H3. Mycobacterial infection changes genome-wide DNA methylation. The host cell can modify the expression of epigenetic effector proteins like DNA and histone methyltransferases to activate immune-responsive genes, prevent mycobacteria from altering its epigenetic profile, or reverse changes in mycobacterial protein epigenetics (5, 32). However, the SUV39H1 methylates the histone-like mycobacterial cell surface protein *HupB*. The lysine methylation by SUV39H1 at K138 of *HupB* occurs in a pattern similar to H3K9, confirming its selective profile (Ku). Mycobacteria possess HNS and HBHA histone-like proteins. SUV39H1 trimethylates a mycobacterial protein and exhibits no pattern (31, 33). Notably, zinc-dependent endopeptidases called matrix metalloproteinases (MMPs) are essential for tissue repair and inflammatory tissue breakdown in diseases like emphysema. The movement of inflammatory cells and the signaling of chemokines/cytokines depend on matrix metalloproteinases. MMP activity strongly affects TB immunopathogenesis (18). MMPs are linked to pulmonary TB and central nervous system immunopathogenesis. A recent study found that histone acetylation regulates MMP-1 and MMP-3 synthesis in response to MTB. This study also found that MTB-infected macrophages had altered HDAC expression patterns, which lower gene expression. Class HDAC gene expression downregulation caused HDAC4 to increase but not HDAC5. However, CoMTb activation did not affect Class I and Class II HDAC expression in pulmonary epithelial cells, suggesting a cell type-specific effect. Toll-like receptors affect histone deacetylase action and expression (18, 34, 35).

Recently, a study evaluated three populations: MDR TB-HIV on ART, HIV-1 not on ART, and uninfected individuals, and results indicated that all three research groups had identical Dicer expression levels according to gene expression data. Dicer protein processes microRNAs that regulate gene expression, but it hasn’t been linked to epigenetic modifications, making this conclusion expected. *TET1* and *DNMT1* gene expression was lower in MDR TB-HIV patients, moderate in uninfected individuals, and high in HIV-1 positive patients. The *HAT* gene, which codes for the HAT enzyme involved in histone acetylation, along with HDAC for histone deacetylation and the *PtkA* and *PtpA* genes for phosphorylation and dephosphorylation, showed significant upregulation in HIV-1 patients, moderate upregulation in MDR TB-HIV co-infected individuals, and significant downregulation in healthy controls. HAT acetylates histones, allowing access to chromatin and gene promoters (36).

### 3.3 Epigenetics links to non-coded RNAs

Long non-coding RNAs (lncRNAs) modulate gene expression to orchestrate the immune response. These RNAs regulate immune cell functioning, physiological processes, and infectious responses (37). Also, small non-coding RNAs (sRNAs) control mRNA stability, processing, and ribosome-binding site access in mycobacteria. sRNAs are essential for bacterial survival in stressful conditions, such as host immune response (38–40). MTS1338, a conserved sRNA in pathogenic mycobacteria, accumulates in dormant MTB cells and is upregulated in mycobacteria phagocytosed by macrophages *in vitro* and in mouse infection models, indicating its role in host-pathogen interactions. However, the study found that about half of the DEGs in the stressed MTS1338-overexpressing strain *in vitro* matched those activated in MTB within peritoneal macrophages: 32 of 60 DEGs under normal conditions, 32 of 63 at low pH, 16 of 42 with ROS, and 54 of 83 with RNS. The findings suggest that MTS1338 overexpression activates adaptive mechanisms for mycobacterial survival in the harsh intra-macrophage environment during MTB infection. However, the MTB transcriptome profiles in this study were compared to those in the peritoneal macrophages of I/St mice to interpret the results (13, 41).

Besides, miRNAs control gene expression after transcription and play a role in immune regulation, inflammation, and cellular stress. MicroRNAs control immunity to MTB. *MiR-21, miR-155, and miR-146a* regulate T cell differentiation, autophagy, cytokine synthesis, and macrophage activation. However, *hsa-miR-15a-5p* had the highest increase in corrected p-values, suggesting its potential as a biomarker and regulator in TB pathogenesis (42). *miR-15a-5p*, part of the miR-15/16 family, affects apoptosis, regulates the cell cycle, and impacts inflammatory cytokine production. *MiR-15a-5p* influences pro- and anti-inflammatory responses by regulating genes in the BCL2, IKKα, and NF-κB pathways (43). TB host defense requires miR-15a-5p to regulate autophagy, apoptosis, cytokine production, macrophage activation, and polarization. In contrast, genes like *mTOR, ET-1, YAP, CXCL11, CD44, MAP3K4, p65, IKKα/β, ICAM-1*, and *p85α* are regulated by *miR-15a-5p* or *miR-199a-3p*, influencing atherosclerosis, lipid metabolism, and inflammation. These miRNAs are linked to inflammation, including NF-κB pathway proteins, based on in silico experiments and literature analysis. IKKα, IKKβ, and p65 were identified as potential options (44).

Additionally, competitive endogenous RNA (ceRNA) like lncRNA and circRNA compete with mRNA for target gene expression-regulating miRNA response elements (MREs). DEcircRNA research indicated that downregulated circRNAs were substantially more numerous than upregulated ones and all were downregulated in the core circRNA–miRNA–mRNA network, suggesting they may be important in pulmonary TB pathogenesis. In TB patients, *has-miR-607* interacts with downregulated circRNAs such *hsa_circ_0000566* and *hsa_circ_0005408*, upregulating its expression and altering IFNG expression Several immune system activities are affected by low core regulatory network IFNG expression (45).

On the other hand, the LC3 autophagy marker. Autophagosome closure needs cytoplasmic LC3 (LC3-I) to break down a short polypeptide fragment and form an autophagic membrane (LC3-II). LC3-II/I indicates autophagy levels. Western blotting and immunofluorescence detected alterations in the LC3-II/I ratio and puncta. In this context, *miR-1958*’s role in autophagy control. In RAW264.7 cells, *miR-1958* enhances siRNA silencing. Autophagy is dynamic and influenced by various factors, making it essential to study Atg5’s relationship with other autophagy genes. Immunofluorescence tests showed that the *miR-1958* mimic reduced autophagosomes, while the inhibitor increased LC3 puncta. MTB blocks autophagy to safeguard the immune system (46). Significantly, patients with active tuberculosis exhibit elevated DNA methylation of the LC3B gene promoter. MTB modulates autophagy by epigenetic alterations to ensure survival. Especially. host *miR-30a, miR-155*, and *miR-1303* could suppress MTB-induced autophagy by targeting ATG3 and ATG2b. MTB suppresses autophagy in host cells by hyper-methylating histone H3 lysine 9 at the promoters of the Atg5 and Atg7 genes via p38-MAPK and EHMT2 methyltransferases (14, 47). Moreover, the LC3B promoter DNA methylation levels were higher in active TB patients than in LTBI or non-infected healthy subjects, but they remained unaltered following anti-TB medication. As neither Atg5 nor the LC3B gene promoter was altered by ESAT-6 stimulation *in vitro*, the hypermethylation may be an epigenetic predisposition to active tuberculosis rather than a modification caused by chronic MTB infection (48).

### 3.4 Advanced tools to analyze the epigenetic mechanisms

Insertion of the MTB *Rv1515c* gene into *M. smegmatis* altered its shape and surface topology. The colony shape and structure of M. s_Rv1515c may be influenced by cell surface features. Mycobacteria may have *Rv1515c* on their membranes and walls. *Rv1515c* regulates lipids like 7-heptenyl-lauric acid (C12:1ω7c), 3-methyl-hexadecanoic acid (3MeC16:0), nonoic acid (C9:0), and undecanoic acid (C11:0). This is linked to changes in mycobacterial cell envelope lipids, cell surface hydrophobicity, and cell wall shape, as noted in a previous study by Yang et al. *Rv1523* and mmaA4 of MTB affect mycolic acids in the cell wall (49, 50). Recombinant M. s_Rv1515c cells were absorbed more by macrophages than control cells. M. s_Rv1515c’s higher invasiveness suggests pathogenicity. M. s_Rv1515c cells stayed in macrophages for up to 72 hours, unlike control M. s_Vc cells. MTB can survive in macrophages and lead to latent infections because of *Rv1515c* (51, 52).

Subsequently, four cysteine residues in *Rv1377c.* that may bind Zn²^+^, and isothermal titration calorimetry (ITC) tests revealed that Zn²^+^ binding to protein decreases S-adenosylmethionine (SAM). Also, circular dichroism spectroscopy (CD) and proteinase K cleavage studies clarify this. This concept proposes that Zn²^+^-mediated enzyme inhibition influences metabolism and signal transmission, alongside its catalytic and structural roles. However, *Rv1377c* is a unique methyltransferase controlled by the SOS response in MTB. The SOS response is an effective mechanism used by bacteria to handle stress. This indicates that *Rv1377c* might also aid in MTB’s survival under stress (53). In addition, the role of IL-1 is in β-glucan-mediated protection in human monocytes; β-glucan induces trained immunity effectively. However, a recent study analyzed ChIP-seq and genome-wide RNA sequencing data from β-glucan-exposed human monocytes. β-glucan affects genes crucial for the IL-1 signaling cascade. Epigenetic changes from β-glucan increase the production of IL-1 family members. β-glucan therapy boosts host defense and survival through epigenetic changes in monocytes and macrophages, resulting in improved recruitment prior to the MTB infection (54).

### 3.5 Host direct therapy and future perspective

**Figure 2:**
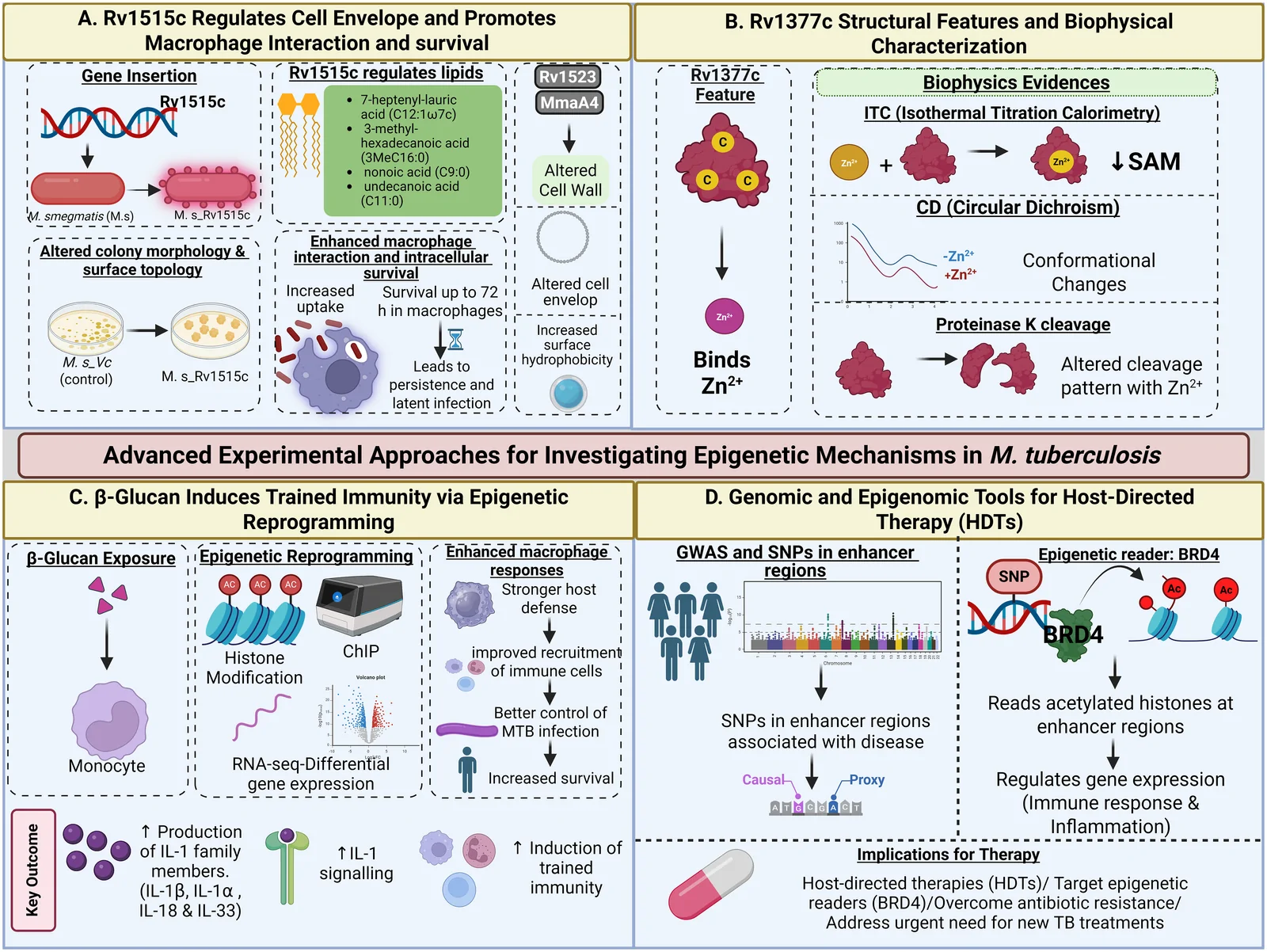
shows experimental data and new methods for studying epigenetic regulation in Mycobacterium TB infection. summarises key experimental results and cutting-edge methods for examining epigenetic regulation during Mycobacterium TB infection. **(A)** Rv1515c increases macrophage absorption, modifies colony architecture and cell wall characteristics, controls the lipid composition of the mycobacterial cell envelope, and encourages intracellular survival and persistence. **(B)** Using isothermal titration calorimetry (ITC), circular dichroism (CD) spectroscopy, and proteinase K cleavage experiments, structural and biophysical characterisation of the methyltransferase Rv1377c shows Zn²^+^ binding and related conformational changes. **(C)** Through epigenetic remodelling, β-glucan-induced trained immunity reprograms monocytes, leading to histone alterations, changed gene expression, enhanced macrophage antimicrobial responses, higher production of IL-1 family cytokines, and improved control of Mtb infection. **(D)** Genome-wide association studies (GWAS), enhancer-associated single nucleotide polymorphism (SNP) analysis, and epigenetic reader proteins like BRD4 are examples of genomic and epigenomic approaches that offer mechanistic insights into host-directed therapies (HDTs) intended to modify immune responses and overcome antibiotic resistance in tuberculosis.

The genome-wide association studies have connected single nucleotide polymorphisms (SNPs) in gene enhancer regions to specific diseases. These findings emphasize the need to target epigenetic mechanisms such as bromodomain containing proteins (BRD4), which reads these SNPs, for disease treatment. Host-directed treatments (HDTs) are gaining importance due to the rapid increase in antibiotic resistance and the decrease in new antibiotic discoveries. Strategies targeting these characteristics could enhance tuberculosis therapy (**Figure 2**) (55).

Furthermore, an *Rv3034c* encodes an acetyltransferase. MTB genome studies indicated that *Rv3034c* is essential for mycobacterial survival and function. *Rv3034c*, a cell-wall protein, may mediate host–pathogen interactions. The Mtb::Rv3034c(+pptr) strain improved intramacrophage survival, indicating that *Rv3034c* is essential for mycobacterial survival in macrophages. Eis protein, arylamine N-acetyltransferase, and MTB acetyltransferase help MTB survive in macrophages (56). Noticeably, epigenetic regulator *Rv3423.1* alters anti-inflammatory genes to help bacterial survive, Eis redox-dependently acetylates histone H3, activates the Akt/mTOR/p70S6K pathway, and regulates autophagy, inflammation, and cell death to increase IL-10 and destroy host innate immunological defenses. It produces L-cysteine precursor OAS. O acetylserine (OAS) reduction in the *ΔcysE* mutant may result in decreased L-cysteine production and lower antioxidant levels, such as free thiols. This would increase MTB oxidative stress and decrease survival *in vivo* (57, 58).

### 3.6 Clinical and translational relevance of epigenetic modulation in tuberculosis

DNA methylation in BCG vaccination was analyzed by limited studies. Consequently, the BCG responder-associated module prominently features p75NTR pathway interaction proteins. This pathway is implicated to regulation of Rac and Cdc42. Research on the p75NTR pathway in phagocytic uptake is limited, but indirect evidence indicates it may influence phagocytosis (59). A recent bioinformatic study found that BCG responder macrophages phagocytosed mycobacteria more effectively than non-responders. Differentially methylated genes (DMGs) regulate phagocytosis; IL-1β production is unaffected by methylation and occurs later. The correlation between β-values of 43 DMGs and phagocytosis is strong; IL-1β has a weaker correlation. IL-1β is produced to fight intracellular infections, usually following microbial invasion or phagocytosis. Phagocytic uptake may reflect the effectiveness of IL-1β production (60).

Moreover, few studies exist on epigenetic modulation and its connection to differentially expressed genes. Notably, the SIRT2 inhibits stress-induced pro-inflammatory cytokines and surface activation markers in several cell types. Bhaskar A et al. found that AGK2 treatment increased mice’s peritoneal macrophage activation, T cell activation, and proinflammatory cytokines following MTB infection (61). Similarly, Cheng et al. observed reducing AGK2 and SIRT2 increased acetylation of histone H3K18 and NFκB p65 in MTB-specific T cells, leading to increased proinflammatory gene expression. SIRT2 inhibition promotes T cell Th1/Th17 responses during TB development (62). Particularly, intracellular infections like *Anaplasma phagocytophilum, Pseudomonas aeruginosa*, and *Porphyromonas gingivalis* upregulate HDACs, which epigenetically inhibit host defense genes. However, pathogens target sirtuins to change the host acetylome. *Salmonella typhimurium* increases SIRT2 to regulate NOS2 and degrades SIRT1 to inhibit autophagy (63, 64). Furthermore, activation of SIRT1 boosts autophagy and phagosome-lysosome fusion (12), aiding MTB survival. MTB infection significantly increases SIRT2 levels in peritoneal macrophages for the first time. After siRNA treatment, AGK2’s suppression of SIRT2 and the decrease in SIRT2 reduced MTB’s intracellular survival. Although, phase 2 human studies are underway for safe SIRT1 activators. As TB HDT, they may prevent resistance, shorten multidrug TB treatment, and enhance immune responses to eliminate pathogens. SIRT1 activators may alleviate tuberculosis-related lung issues by moderating chronic inflammation. Exploring SIRT1 activators for TB treatments requires overcoming several challenges. Recent research does not address the impact of SIRT1 activators on the adaptive immune response that controls MTB infection. The role of SIRT1-deficient myeloid cells in causing TB is unclear. Research on macaques and next-gen SIRT1 synthetic activators is vital (62).

## 4 Limitation of TB epigenetics

The main constraint of tuberculosis epigenetics is the lack of mechanistic understanding, applicable models, adequate host lung disease data, technological limitations, and a shortage of epigenetic-based interventions for TB. The upcoming technological tools will tackle these challenges, such as single-cell epigenomics, host lung tissue studies, multi-omics methods, and validation in clinical translational research. However, recent studies explored alveolar macrophages’ (AMs’) trained immunity, while the foundational work used PBMC. Intradermal BCG assessments before and after immunization in human AMs showed no trained immunity. Two weeks and three months post-immunization, induced sputum AM showed unchanged inflammatory cytokines and reduced CD11b and MHC-II expression (65). Studies show that the BCG distribution route is vital for the immune response, making these negative results clear. HDAC inhibitors can reverse histone changes in MTB infection by regulating IL-1β and IL-10 at the histone level. RGFP9966’s HDAC3 inhibition showed the strongest antibacterial effect on MTB-infected AM. Our AM experiments indicate that MTB infection causes chromatin changes, leading to significant transcriptional modifications that impact the immune response. DNA methylation controls MDM and AMPK signaling pathways and THP-1 IL-6R, IL-4R, and IL-17R. Though all of these studies employed cell lines and blood-derived macrophage models, they all showed that epigenetic mechanisms reduce host immunity to MTB infection. Thus, adequate lung tissue data should be required to fill the research gap (66, 67).

## 5 Discussion

This review analyses the epigenetic mechanisms in MTB that counteract host immune responses. Multiple genes and enzymes help MTB defend itself and survive in a dormant state within macrophages. Pathogen-induced epigenetic changes may affect mycobacterial survival molecules (65). HDAC2 downregulates Ehmt2 (G9a), activating NEDD4-family ligases. Protein arginine methyl transferases (PRMTs) play a role in infections and E3 ligase regulation, yet research on them is limited (68). Epigenetic regulators are associated with the progression of various infectious diseases, such as TB. PRMT5 and PRMT9 mediate symmetric arginine methylation, while PRMT1, 2, 3, 6, 8, and CARM1 mediate asymmetric methylation (69, 70). The MTBC’s evolutionary groupings’ methylation patterns are convergent. Some genetic variants cause comparable symptoms (non-methylation of motifs). E270A in certain L2 strains affects *MamA* activity like W136R in some L1 strains. Methylation appears to have minimal influence on *in vitro* gene expression. The study found no regulatory effect on major methyltransferases except for a few genes with overlapping *SigA* and *MamA/MamB* recognition motifs. Differential expression of genes unrelated to *HsdM* in the *Δhsdm* strain may hamper identification of methyltransferases-regulated genes (23, 27).

Further study is needed on how MTB infection controls host cholesterol process. Epigenetic processes, including miRNAs (*miR-33a, miR-185*), histone deacetylases (HDAC3, SIRT2, SIRT6), and HMTs (G9a), regulate cholesterol production and homeostasis genes. MTB infection relies on sirtuins. SIRT3 boosts antimycobacterial defenses. SIRT2 decreases MTB burden, while nuclear SIRT1 boosts autophagy to inhibit MTB production. SIRT1 influences ageing, stress, anti-inflammatory response, and lipid metabolism. SIRT6 levels were found to be elevated in MTB infection, linked to transcriptional analysis (71, 72).

FOXO3 decreases inflammation, enhances antimicrobial peptide synthesis, suppresses T-cell activation, cytokine production, and NF-κB activation. *MiR-223*’s MTB-induced macrophage apoptosis inhibition is counteracted by FOXO3. TB patients with hypomethylated FOXO3 at the gene body had a higher all-cause death rate one year after enrolment, possibly due to its participation in anti-TB immune responses. A unique negative regulator of LPS-induced inflammation, GNG12 reduces nitrite and TNF-α levels. Hypermethylated GNG12 epigenotype may impair host immune responses, creating MTB infection risk and worse outcomes (73, 74). In addition, individual resistant to TST/IGRA conversion (RSTR) showed lipid-associated voltage-gated potassium channel KCNQ1 methylation patterns. Various mutations in KCNQ1 cause plasma lipid buildup, whereas KCNQ1OT1, the antisense long non-coding RNA of KCNQ1, increases lipid accumulation in macrophages and reduces cholesterol efflux ability. Lipid transport and lipase affect free fatty acids (FFA) levels and lipid buildup. Foamy or lipid-rich macrophages may help MTB survive (75, 76).

In immune response against mycobacterial infection, the IL-10 gene-deficient mice have increased Th17 and Th1 responses to MTB, highlighting IL-10’s regulatory role. This aligns with increased MTB regulation in chronic infection. Other cytokines, like TNF-α, are crucial for controlling mycobacterial growth, granuloma formation, and starting the adaptive immune response to MTB, thus protecting the host. A prior investigation showed that MTB Beijing/W strains significantly increased the production of interleukin-6 (IL-6) and interleukin-1β (IL-1β) in dendritic cells. Cytokines are crucial for protection against mycobacterial infections (16). MamC methylation may impact *GuaA* expression, which may benefit GuaA-targeting therapies. At –25 bp, *Rv1219c*’s *raaS* promoter has the *MamC* motif. The transcriptional regulator *RaaS* overexposes the ABC efflux pump proteins *Rv1217c–Rv1218c*, increasing the minimum inhibitory concentration for isoniazid and rifampicin, the first line anti-tuberculosis drugs after *MamC* deletion, *raaS*’s promoter *MamC* motif indicated significant transcriptional alterations. *Rv1217c–Rv1218c* efflux pump activity may impact drug tolerance if *MamC-*induced *raaS* promoter methylation directly affects *raaS* expression (26).

Mycobacterial species evade the host immune system using unique strategies (**Table 2**). Efflux inhibitors are being studied as adjuvant therapy in preclinical research due to the resistance mechanism of efflux pumps. Presumptive treatments for mycobacteria include synthetic and natural efflux inhibitors. Host immunological responses are important but rarely examined for efflux inhibitors (79, 80). In addition, host colonization of MTB utilizes the lipid-rich environments; however, cholesterol binds to *CeoB*, a component of the MTB Trk K^+^ uptake system, influencing its activity and host colonization. However, cholesterol controls bacterial biology and supplies carbon. Cholesterol can affect K^+^ transport in animals by binding to K^+^ transport pathways, either increasing or decreasing the transport (77, 78, 81).

**Table 2:** MTB evasion mechanisms and epigenetics roles.

Study	Epigenetic/transcriptomic marker	Mechanism of action	Study outcome	Technical platform
Zhang et al., 2025 (77)	MAB_2885; MAB_2302 and MAB_2303	Misfunctioning MAB_2885 caused TZD resistance. Comparing the MAB_2885-supplemented group to the control group (T1 and T7 with empty pMV261BL plasmid) using RNA-seq. The log2FC values of 11 significantly up-regulated and 11 down-regulated genes ranged from -4.15 to 1.28. MAB_2302 and MAB_2303 were down-regulated 5.78 and 7.39 times, respectively. Additionally, Intracellular TZD concentrations were measured by LC-MS/MS to investigate if MAB_2302-MAB_2303 and its regulator MAB_2885 alter TZD accumulation.	These results indicate that MAB_2885 inhibits MAB_2302-MAB_2303, the sole TZD-resistant operon. Also, MAB_2885 mutant strain T1 and MAB_2302-MAB_2303 overexpression strains revealed significantly reduced intracellular TZD accumulation, indicating a TZD efflux pump function.	RNA-seq; LC-MS/MS
Chen et al., 2025 (78)	MceG; K+; KstR1	The MceG Mtb ATPase helps the Mce1 and Mce4 transporters import cholesterol and fatty acids. In Mtb growth on cholesterol medium, higher intrabacterial [K+] levels boost MceG activity, promoting cholesterol absorption and use. MceG produced ATPase activity as expected, whereas KstR1, a transcription factor that regulates cholesterol regulon gene expression, did not. Intrabacterial K+ is hundreds of millimolar.	higher [K+] increased MceG ATPase activity. A different cation, sodium, did not influence MceG ATPase activity.	*In-vitro* assay. Cholesterol medium
Behera et al., 2022 (56)	Rv3034c as Acetyltrasferase activity	Mtb-infected bone marrow derived macrophages (BMDM) had increased peroxisomal catalase. QRT-PCR demonstrated that Mtb-infected macrophages with Rv3034c had substantially higher catalase levels than those without. Msm-Rv3034c-infected macrophages expressed more transcriptional and translational catalase.	Msm-Rv3034c-infected macrophages expressed more transcriptional and translational catalase. a potential ROS scavenger.	qRT-PCR
Zhang et al., 2025 (57)	Rv0819, Rv1347c, and Rv2335	M. tb lung infection needed three genes (Rv0819, Rv1347c, and Rv2335) on day 14 and four on day 28.and tb survival in mice our screening strategy. Many host macrophages stresses stress M. tb. affecting ΔcysE’s survival *in vivo*The ΔcysE mutant reacts normally to dietary restriction, acidic pH (5.5), and SDS-induced membrane stress. However, ΔcysE exhibited a 10-fold lifetime reduction and increased susceptibility to all peroxide stressors (H2O_2_, MND, and CHP) when exposed to radicals. This survival gap was somewhat compensated by OAS	TU, a ROS scavenger, improved ΔcysE’s growth and survival in 7H9 medium and under peroxide stress. Results suggest ROS sensitivity is the main cause of ΔcysE mutant’s *in vitro* growth and survival issues.	*In-vitro* THP1 assay
Prakhar et al., 2023 (72)	G9a and SIRT6	Filipin staining demonstrated that *in vitro* siRNAs reduced Ehmt2 and Sirt6 reduce MTB H37Rv cholesterol. siRNAs to perform the MTT test at 48 hpi with and without G9a or SIRT6 knockdown to track cell viability changes produced by siRNA on infection. Unaffected cell viability. cholesterol kit to confirm Filipin staining showing MTB infection raises cholesterol via G9a and SIRT6. The cholesterol estimation based on cell population (4 million each) was normalised to total cellular protein to exclude cell survival effects. Silencing G9a and SIRT6 lowers H37Rv-induced cholesterol.	In severe Mtb H37Rv infection, host macrophages collect cholesterol, unlike M. smegmatis. Filipin staining of lung cryosections demonstrated elevated cholesterol levels in Mtb H37Rv-infected macrophages. G9a and SIRT6 epigenetically affect cholesterol levels	Filipin staining; MTT assay

Importantly, small RNAs play a key role in regulating the host immune response and the survival of mycobacteria. Also, lung commensals and links to non-coded RNA functions are important to evaluate combined with epigenetics in MTB infections, which is crucial. However, a lncRNA-CGB (commensal gut bacteria) inhibits H3K27Me3 changes in the IFN-γ promoter, boosting IFN-γ production and offering protective effects during MTB infection. lncRNA-CGB binding to EZH2 did not directly impact PRC2’s enzymatic activity. This binding may impede EZH2’s genomic targeting at the *ifng* promoter, weakening its effects and lowering its suppressive role (82). The aforementioned analysis of MTB linked to epigenetic modification is vital for the survival of mycobacterium, and more research on these genes will be critical for comprehending their significance in therapy. Projections on the advancement of therapies targeting microbe-host interactions. Additionally, multiple genes were analyzed for clinical relevance of the infections; particularly, prominent features include the p75NTR pathway, SIRT2 inhibits stress-induced pro-inflammatory cytokines and surface activation markers, and AGK2 and SIRT2 increased acetylation of histone H3K18 and NFκB p65 in MTB-specific T cells. Thus, focusing on these pathways and markers will enhance the analysis of drug-resistant and drug-susceptible TB infections.

## 6 Conclusion

This review examined the functions of epigenetics in MTB infections and how they relate to the immunological responses of the host. Still, there are multiple factors connected to epigenetic regulation; one of the most understudied is how MTB responds to host interactions via epigenetic studies and the role of non-coding RNAs. However, there are numerous small RNAs, such as lncRNAs and circRNAs, with regulations linked to the prognosis of the TB infection. Thus, transcriptome analysis of microbe-host interactions plays a vital role. In addition, recent emerging technologies such as CRISPR, SNP, WGS, and multi-omics models are advancing the therapeutic target against TB infection. Therefore, further studies are needed to determine the major role of small RNAs in epigenetic functions and develop host-directed therapeutic targets against TB infection.
